# 3D Compartmentalised Human Pluripotent Stem Cell–derived Neuromuscular Co-cultures

**DOI:** 10.21769/BioProtoc.4624

**Published:** 2023-03-05

**Authors:** Peter Harley, Amaia Paredes-Redondo, Gianluca Grenci, Virgile Viasnoff, Yung-Yao Lin, Ivo Lieberam

**Affiliations:** 1 Centre for Gene Therapy & Regenerative Medicine, Kings College London, London SE1 9RT, UK; 2 Centre for Developmental Neurobiology and MRC Centre for Neurodevelopmental Disorders, Institute of Psychiatry, Psychology and Neuroscience, Kings College London, London SE1 1UL, UK; 3 Centre for Genomics and Child Health, Blizard Institute, Barts and the London School of Medicine and Dentistry, Queen Mary University of London, 4 Newark Street, London E1 2AT, UK; 4 Centre for Predictive in vitro Model, Queen Mary University of London, Mile End Road, London E1 4NS, UK; 5 Mechanobiology Institute, National University of Singapore, 5a Engineering Drive 1, 117411 Singapore

**Keywords:** Tissue engineering, Neuromuscular co-culture, Compartmentalised microdevice, Human pluripotent stem cells, Optogenetics, Motor neuron, Myofiber, DMD, ALS

## Abstract

Human neuromuscular diseases represent a diverse group of disorders with unmet clinical need, ranging from muscular dystrophies, such as Duchenne muscular dystrophy (DMD), to neurodegenerative disorders, such as amyotrophic lateral sclerosis (ALS). In many of these conditions, axonal and neuromuscular synapse dysfunction have been implicated as crucial pathological events, highlighting the need for in vitro disease models that accurately recapitulate these aspects of human neuromuscular physiology. The protocol reported here describes the co-culture of neural spheroids composed of human pluripotent stem cell (PSC)–derived motor neurons and astrocytes, and human PSC-derived myofibers in 3D compartmentalised microdevices to generate functional human neuromuscular circuits in vitro. In this microphysiological model, motor axons project from a central nervous system (CNS)–like compartment along microchannels to innervate skeletal myofibers plated in a separate muscle compartment. This mimics the spatial organization of neuromuscular circuits in vivo. Optogenetics, particle image velocimetry (PIV) analysis, and immunocytochemistry are used to control, record, and quantify functional neuromuscular transmission, axonal outgrowth, and neuromuscular synapse number and morphology. This approach has been applied to study disease-specific phenotypes for DMD and ALS by incorporating patient-derived and CRISPR-corrected human PSC-derived motor neurons and skeletal myogenic progenitors into the model, as well as testing candidate drugs for rescuing pathological phenotypes. The main advantages of this approach are: i) its simple design; ii) the in vivo–like anatomical separation between CNS and peripheral muscle; and iii) the amenability of the approach to high power imaging. This opens up the possibility for carrying out live axonal transport and synaptic imaging assays in future studies, in addition to the applications reported in this study.

Graphical abstract

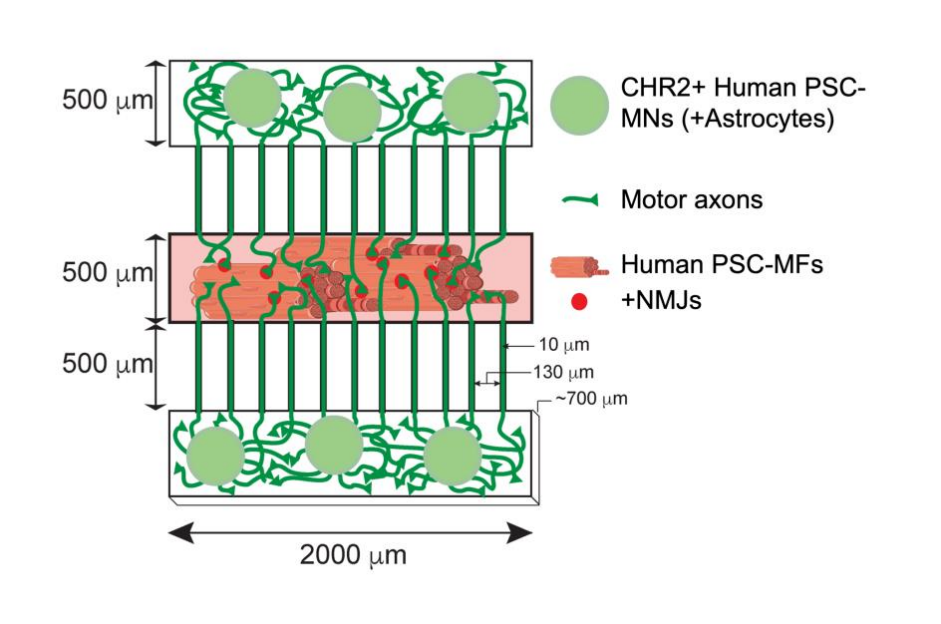

**Graphical abstract abbreviations:** Channelrhodopsin-2 (CHR2+), pluripotent stem cell (PSC), motor neurons (MNs), myofibers (MFs), neuromuscular junction (NMJ).

## Background

Neuromuscular diseases are a diverse group of disorders with considerable unmet clinical need. For instance, many muscular dystrophies, such as Duchenne muscular dystrophy (DMD) that lead to progressive muscle wasting, currently lack effective treatments (Duan et al., 2021). Similarly, neurodegenerative disorders such as amyotrophic lateral sclerosis (ALS), caused by degeneration of motor neurons (MNs) that innervate and control movement of skeletal muscle, also lack effective treatments (Hardiman et al., 2017). Early peripheral phenotypes like axonal and neuromuscular junction (NMJ) dysfunction have been implicated in a wide range of divergent subsets of neuromuscular disorders, including both ALS and DMD (Fischer et al., 2004; van der Pijl et al., 2016). Emerging evidence suggests that therapeutically targeting neuromuscular synapse dysfunction is a viable option for extending lifespan and reducing disease severity in these disorders (Bruneteau et al., 2013; Cantor et al., 2018; Paredes-Redondo et al., 2021). However, a major challenge has been the generation of human-relevant disease models that accurately recapitulate in vivo human neuromuscular physiology and distinct neuromuscular disease phenotypes.

While mouse models have been widely used for studying neuromuscular physiology, growing evidence shows that there are substantial differences between human and mouse neuromuscular synapses, including: contrasting synaptic morphologies, divergent transcriptomes and proteomes, and altered homeostasis and maintenance (Jones et al., 2017).

With the development of differentiation protocols to derive enriched populations of motor neurons and myofibers (MFs) from human pluripotent stem cells (PSCs) (Du et al., 2015; Chal et al., 2016; Fernandopulle et al., 2018; Rao et al., 2018; Cheesbrough et al., 2022; Harley et al., 2022), a number of neuromuscular co-culture platforms have been established. A major challenge for generating human PSC-derived neuromuscular co-cultures has been stabilising contractile myofibers in order to prevent detachment upon contraction and allow long-term culture and maturation (Abd Al Samid et al., 2018). A notable example of an approach that overcame this issue was the development of a microphysiological neuromuscular disease model of ALS by Kamm and colleagues, in which bundles of myofibers were supported between micropillar cantilevers capable of deflecting upon contraction (Uzel et al., 2016; Osaki et al., 2018). Building on this work, we have developed a model of human neuromuscular physiology with a number of innovative features. These include a compartmentalised design comprising three chambers connected by microchannels, which mimics the spatial organization of tissues in vivo. This design enabled us to compare two genetically different motor neuron populations—one sensitive to optogenetic stimulation and the other insensitive—in their ability to innervate the same myofiber targets in response to optogenetic entrainment (Machado et al., 2019). Likewise, it allowed us to study phenotypes of the same motor neurons innervating an isogenic pair of DMD and CRISPR-corrected myofibers (Paredes-Redondo et al., 2021). Furthermore, incorporation of ALS-related TDP-43^G298S^ motor neurons and CRISPR-corrected controls enabled us to recapitulate key ALS-related neuromuscular phenotypes (Harley et al., 2022). In all these experiments, motor neurons were embedded in a scaffold of mouse embryonic stem cell (ESC)–derived astrocytes to promote maturation and provide neurotrophic support. In future studies, it will allow different reconfigurations of the device to support different co-cultures. Such combinations of tissues may include a full corticomotor tract (cortical neurons, motor neurons, muscle) or the incorporation of sensory neurons into a *sensory feedback* compartment. Secondly, the proximity of the individual compartments to the optical polymer allows easy and rapid high-power imaging without the need for additional tissue processing and microdissection. Combined with the microchannels for axonal outgrowth, this would easily allow for high-resolution live axonal transport imaging and live synaptic imaging assays to be developed in future studies. Thirdly, myofibers are stabilized by a thin layer of UV-cured resin applied to the surface of the optical polymer, similar to the anchor points in the original microdevices (Machado et al., 2019). Finally, we provide evidence that formation of functional in vitro human neuromuscular circuits is activity dependent. We found that optogenetic entrainment of the motor neurons over the course of the culture dramatically enhanced synapse formation and myofiber contractility. In future studies, the microdevices described here may be used to understand how different stimulation paradigms and competitive innervation of common synaptic targets facilitates the wiring of human neuromuscular circuits.

## Materials and Reagents

1. 15 mL Falcon tubes (Corning, catalog number: 430791 or equivalent)
2. 50 mL Falcon tubes (Corning, catalog number: 352070 or equivalent)
3. U-bottom 96-well plates (Corning, catalog number: 353227)
4. Silicon master mould (CAD design available in supplementary materials)
5. Channelrhodopsin-2 (CHR2)-YFP+ human PSC-motor neurons (+recommended primary or PSC-astrocytes) (Paredes-Redondo et al., 2021; Harley et al., 2022). Request of relevant cells from the Lieberam and Lin groups are subject to approval of Material Transfer Agreements with the institutions at which they were generated.
6. Human PSC-myogenic progenitors (Paredes-Redondo et al., 2021; Harley et al., 2022). Request of relevant cells from the Lieberam and Lin groups are subject to approval of Material Transfer Agreements with the institutions at which they were generated.
7. Polydimethylsiloxane (PDMS) (Dow Corning, Sylgard-184, catalog number: 4019862); store in the dark at room temperature (RT)
8. NOA-73 (Norland products, catalog number: NOA73); store in the dark at RT
9. Triton X-100 (Thermo Fisher, catalog number: 85111)
10. Growth factor reduced (GFR) matrigel (Corning, catalog number: 356238); store at -20 °C
11. Lipidure (Amsbio, catalog number: CM5206); store 0.5% solution in 100% ethanol at RT in the dark
12. TrypLE (Gibco, catalog number: 12605010); store at 4 °C
13. Accutase (Gibco, catalog number: A1110501); store at 4 °C
14. DNase-I (Roche, catalog number: 10104159001); store at 4 °C
15. Fibrinogen from bovine plasma (Sigma, catalog number: F8630); stock solution 24 mg/mL 0.9% NaCl; store at -80 °C
16. Thrombin from bovine plasma (Sigma, catalog number: 9002-04-4); store at -80 °C
17. Bovine serum albumin (BSA) fraction V (Roche, catalog number: 10735078001), dissolved in D-PBS (Gibco, catalog number: 14190250) to get 5% stock solution, and then sterile filtered. Store at 4 °C
18. 1 M CaCl_2_ solution (Sigma, catalog number: 10043-52-4); store at RT
19. 1,000× antioxidant supplement (Sigma, catalog number: A1345); store at 4 °C
20. DMEM/F-12 (Gibco, catalog number: 11320033)
21. Advanced DMEM/F-12 (Gibco, catalog number: 12634028)
22. Neurobrew-21 (Miltenyi Biotec, catalog number: 130-093-566)
23. Skeletal muscle cell growth medium (M2 in Figure 4) (PromoCell, catalog number: C-23060)
24. N2 supplement (Gibco, catalog number: 17502001)
25. L-glutamine (Gibco, catalog number: 25030149)
26. Penicillin/streptomycin (Gibco, catalog number: 15140122)
27. β-mercaptoethanol (Gibco, catalog number: 21985023)
28. Insulin-transferrin-selenium (Gibco, catalog number: 41400045)
29. GDNF (stock 100 µg/mL; PeproTech, catalog number: 450-10)
30. BDNF (stock 100 µg/mL; PeproTech, catalog number: 450-02)
31. Mouse IgM anti Titin (DSHB, clone: 9D10); store single aliquots at -80 °C
32. Mouse IgG2a anti TUBB3 (R&D Systems, catalog number: MAB1195, clone: Tuj1); store single aliquots at -80 °C
33. Rat IgG anti AChR (DSHB, clone: MAB35); store single aliquots at -80 °C
34. Mouse IgG1 anti-SV2A (DSHB, clone: SV2); store single aliquots at -80 °C
35. Goat anti mouse IgM 405 AlexaFluor (Abcam, catalog number: ab175662); store single aliquots at -80 °C
36. Goat anti mouse IgG2a 488 AlexaFluor (Thermo Fisher, catalog number: A-21131); store single aliquots at -80 °C
37. Goat anti rat IgG 555 (Thermo Fisher, catalog number: A-21434); store single aliquots at -80 °C
38. Goat anti mouse IgG1 647 AlexaFluor (Thermo Fisher, catalog number: A-21240); store single aliquots at -80 °C
39. Vectashield antifade mounting medium without DAPI (Vector Laboratories, catalog number: H-1900-10)
40. ADFNB medium (M1) (see Recipes)
41. Skeletal muscle secondary differentiation medium (M3) (see Recipes)
42. Motor neuron medium (M4); optional (see Recipes)
43. 0.1% PBT (see Recipes)

## Equipment

1. Blunt forceps (Millipore, catalog number: XX6200006P)
2. Vacuum chamber (Thermo Scientific/Nalgene, catalog number: 5305-0609 or equivalent)
3. Vacuum pump (Welch, model: 2522Z-02 or equivalent)
4. Oven for PDMS curing (Binder, model: VD23 or equivalent)
5. UV cross-linker (Vilber-Lourmat, model: BLX-254 or equivalent)
6. Tissue culture hood (Thermo Scientific, model: HeraSafe KS or equivalent)
7. Stereo microscope (Olympus, model: SZX10 or equivalent)
8. Hemocytometer (Neubauer, catalog number: 10360141 or equivalent)
9. Centrifuge (Eppendorf, model: 5810R) with rotor A-4-81 and multi-well plate buckets
10. 35 mm plastic bottom dishes (Ibidi, catalog number: 81156)
11. 15 cm tissue culture dish (Thermo Scientific, catalog number: 353025 or equivalent)
12. 10 cm dish (Corning, catalog number: 430591 or equivalent)
13. Multichannel pipette (Thermo Fisher, catalog number: 4661050N or equivalent)
14. Tissue culture incubator (Thermo Scientific, model: HeraCell 150i or equivalent)
15. Custom LED stimulator for entrainment (Wefelmeyer et al., 2015)
16. 470 nm LED light source (Thorlabs, model: M470F3)
17. LED driver (Thorlabs, model: DC2200)
18. Cell scraper (Falcon/VWR, catalog number: 734-0385 or equivalent)

## Software

1. CellSens (Olympus)
2. Matlab R2021A (Mathworks)
3. PIVLab 2.53 (Thielicke, 2014)
4. IMARIS 9.1.2 (Oxford Instruments)

## Procedure

- **Preparation of compartmentalised microdevices (Figure 1, Video 1)**
  **Figure 1.**
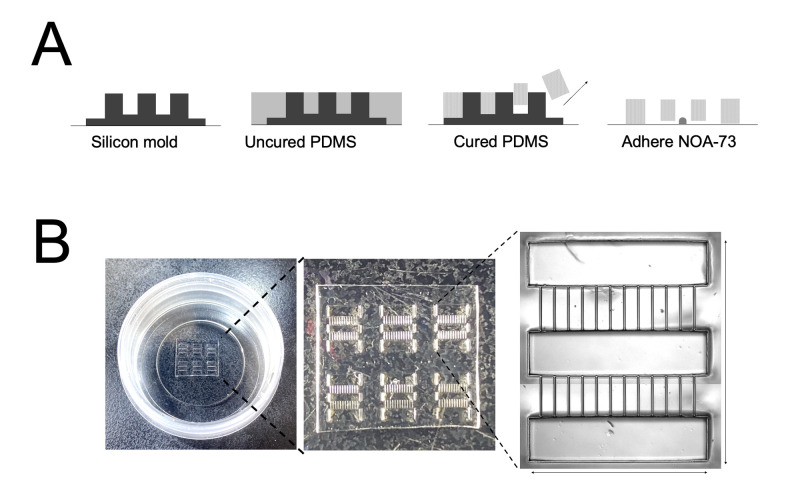
Manufacture of compartmentalised polydimethylsiloxane (PDMS) microdevice arrays in 35 mm tissue culture dishes. **A.** Schematic showing the steps for generating microdevice arrays by curing PDMS on a silicon master mould and adhering the arrays onto 35 mm tissue culture dishes using the UV-curable polymer NOA-73. **B.** Pictures showing the final microdevice array in the 35 mm tissue culture dishes.
  **Video 1.**
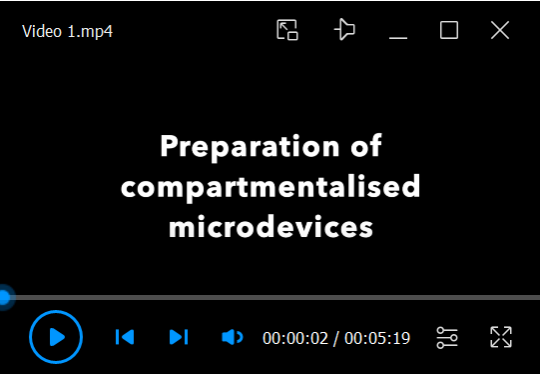
Preparation of compartmentalized microdevices
  1. Mix 2 g of PDMS base 10:1 with curing agent in a 50 mL Falcon tube by pipetting for 1 min and apply uniformly across the silicon master mould. Avoid introducing air bubbles during mixing.
  2. Degas in a 4.7 L vacuum chamber at 800 psi for 20 min.
  3. Cure at 80 °C in the oven for 2 h.
  4. Carefully peel the PDMS wafer using blunt forceps and place into a sterile 15 cm^2^ tissue culture dish microchannel side down to prevent debris from accumulating inside them.
  5. Use a scalpel to cut wafer into desired arrays (2 × 3 or 3 × 3 device arrays work well for a 35 mm dish).
  6. Apply 0.5 μL of the UV-curable polymer NOA-73 to a plastic bottom 35 mm dish and spread evenly using a cell scraper. Mark on the side of the dish the orientation of the final spread since myofibers align better along the same direction as the ridges in the polymer.
  7. Partially UV-cure at 55 J/cm^2^ for 10 s in a UV crosslinker.
    Caution: Partial curing is important to increase the viscosity of the resin while still retaining adhesiveness. If the UV exposure is too short, the resin will fill the microgrooves. If it is too long, the PDMS will not adhere to the dish.
  8. Transfer a 2 × 3 or 3 × 3 array onto the 35 mm dish ensuring that the long axis of the compartment follows the direction of the NOA-73 spread.
  9. Fully UV-cure the device at 55 J/cm^2^ for 1 min on each side.
  10. UV sterilize under UV lamp for 20 min.
  11. Apply 1 mL of 1:50 GFR matrigel in DMEM/F12 to the dish.
  12. Under a stereomicroscope placed in a tissue culture hood, use a fine pipette to remove air bubbles from each large compartment.
  13. Degas the microchannels in a vacuum chamber at 800 psi for 2 h.
  14. Use immediately or seal culture dish with parafilm and store at 4 °C for up to two weeks.
- **Generating neural spheroids**
  This protocol is designed to work with multiple human PSC differentiation protocols. The human PSC-motor neuron differentiation protocol used for this study is from Zhang and colleagues (Du et al., 2015) with additional magnetic-activated cell sorting purification steps outlined in (Machado et al., 2019). Astrocytes (ACs) were derived from mouse ESCs as described in (Machado et al., 2019). Other notable protocols for generating human PSC-motor neurons and astrocytes include (Julia et al., 2017; Canals et al., 2018; Fernandopulle et al., 2018). For the protocol used for these experiments, follow those outlined in (Harley et al., 2022). Consult the original protocol to determine the best point to dissociate and replate the motor neurons/astrocytes.
  1. To generate non-adherent 96-well plates neural spheroids, add 100 μL of 0.5% lipidure dissolved in 100% ethanol absolute to each well of a U-bottom 96-well plate.
  2. Leave plates near the air vents of the tissue culture hood with the lids ajar to dry overnight.
  3. The next day, dissociate motor neurons and astrocytes into single cells using TrypLE or accutase, respectively. For motor neuron dissociation choose a stage in the protocol that allows live cell sorting (Harley et al., 2022) and replating into different culture formats; if motor neurons are too mature with large axonal projections, then dissociation will result in cell death. Astrocyte dissociation may require longer incubations (5–20 min) and addition of DNase-I to prevent cell clumping.
  4. Count the cells using a hemocytometer and plate 15,000 motor neurons and 5,000 astrocytes in each well of the lipidure plate. Calculate the quantity needed for a full plate and use a multichannel pipette. Plate into ADFNB medium. [Optional]: Combine ADFNB medium 50:50 with motor neuron medium. We have successfully used both medium conditions for human PSC-motor neuron spheroid formation.
  5. Briefly spin the plates at 1,200 rpm (290 *× g*) for 2 min.
  6. After 48 h, neural spheroids of uniform size and cell type composition should have formed. Check that the edges are well defined and the overall morphology is round (Figure 2).
    **Figure 2.**
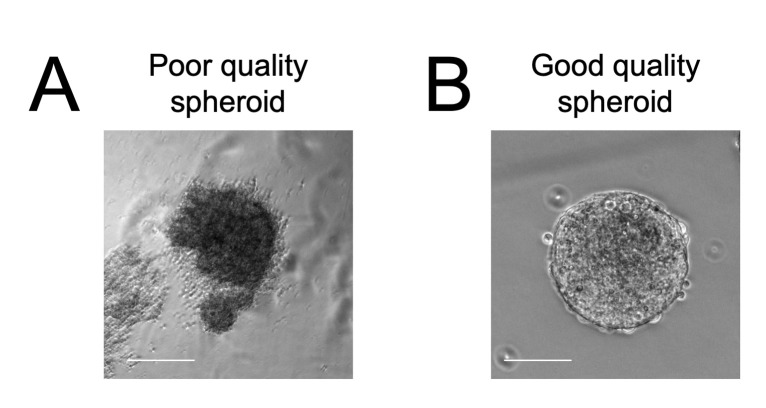
Examples of poor (A) and good quality (B) motor neuron (MN)/astrocyte (AC) spheroids. Scale bar = 50 μm.
- **Plating neural spheroids into the compartmentalised microdevices (Figure 3)**
  1. Thaw GFR matrigel, fibrinogen, and thrombin on ice. Place metal tube rack, metal block, and 35 mm dish on ice to cool.
  2. Make hydrogel mix containing 160 μL of fibrinogen, 40 μL of GFR matrigel, and 0.5 μL of CaCl_2_ (1 M stock) and leave on ice.
  3. Use a multichannel pipette to transfer neural spheroids from the U-bottom 96-well lipidure plates to a 10 cm dish containing 5 mL of ADFNB medium. Pipette up and down several times to dislodge the spheroids but do not be too vigorous as it risks breaking up the spheroids.
  4. Swirl the plate and transfer all the spheroids in 1 mL of medium to a 15 mL Falcon tube.
  5. Remove 950 μL of medium and add 50 μL of hydrogel mix so all the neural spheroids are in 100 μL of 50:50 medium to hydrogel.
  6. Transfer these spheroids to the pre-cooled 35 mm dish and leave on the cool metal block to prevent hydrogel polymerization.
  7. Remove GFR matrigel from the compartmentalised microdevices in the 35 mm dishes (from step A). Directly apply the aspirator to the PDMS array to remove medium from the chambers.
  8. Add 500 μL of ADFNB medium to the edge of the 35 mm dish to prevent drying. Ensure that this does not infiltrate the compartmentalised microdevice array.
  9. Using a fine pipette tip to transfer three neural spheroids to each outer chamber of the microdevice array under a sterile hood microscope.
  10. After completing the whole array, leave to rest for 5 min at RT.
  11. Add extra hydrogel mix to each chamber to ensure there is a meniscus of hydrogel above the PDMS array (this ensures that addition of thrombin does not disturb the neural spheroids).
  12. Now, add ~30 μL of thrombin across the compartmentalised PDMS array to convert fibrinogen to fibrin and initiate polymerisation. Do not add directly to the chambers; just apply at the corners. Do this relatively fast as the thrombin acts quickly to polymerise the hydrogel.
  13. Leave to rest at RT for 5 min.
  14. Now, carefully add 2 mL of ADFNB medium to the dish and place in the incubator.
- **Plating myogenic progenitors into the compartmentalised microdevices (Figure 3)**
  **Figure 3.**
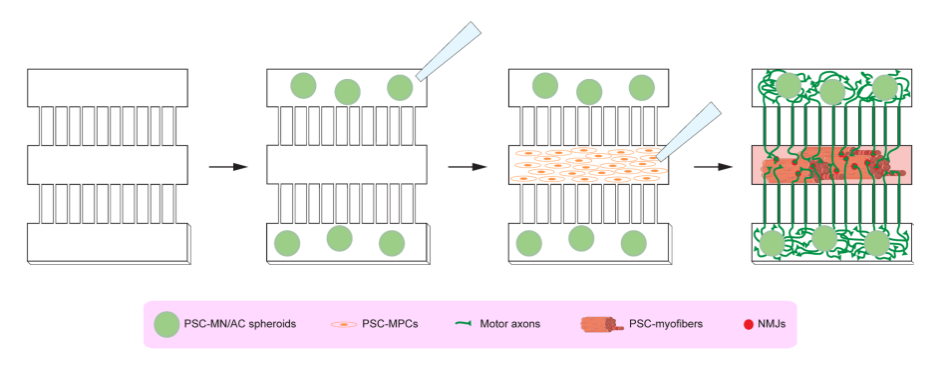
Schematic outlining the different steps for plating human pluripotent stem cell (PSC)–neural spheroids and human PSC-myogenic progenitors into the compartmentalised microdevices. MN: motor neuron; AC: astrocyte; MPCs: myogenic progenitor cells; NMJ: neuromuscular junction.
  This protocol is designed to work with multiple human PSC-myogenic differentiation protocols. The one used for this study is from Pourquie and colleagues (Chal et al., 2016), although similar results were also obtained using forward programming of human PSCs by forced expression of PAX7, as described in (Rao et al., 2018; Cheesbrough et al., 2022). Cells should be replated when they are at an equivalent progenitor/myoblast stage, before myotube fusion has occurred.
  1. After plating the neural spheroids, wait for 24 h before plating the myogenic progenitors. During this time, motor axons will grow through the microchannels into the central compartment.
  2. Thaw GFR matrigel, fibrinogen, and thrombin on ice. Place metal tube rack and metal block on ice to cool.
  3. Make hydrogel mix containing 160 μL of fibrinogen, 40 μL of GFR matrigel, and 0.5 μL of CaCl_2_ (1M stock) and leave on ice.
  4. Dissociate myogenic progenitors into single cells using TrypLE or accutase.
  5. Count cells and resuspend at a concentration of 20,000 cells/μL.
  6. Mix myogenic progenitor suspension with hydrogel mix 50:50, so that cells are now at a concentration of 10,000 cells/μL, and keep on ice.
  7. Remove medium from the edge of the 35 mm dish containing the neural spheroids plated in the outer compartments of the microdevice array.
  8. Add 500 μL of skeletal muscle growth medium to the edge of the 35 mm dish to prevent the neural spheroids in the compartments from drying. Ensure that the medium does not spill onto the microdevice array in the centre of the dish.
  9. Under the sterile hood microscope, use a fine pipette tip to remove residual medium from the empty central chambers of each microdevice.
  10. Now transfer ~1 μL of myogenic progenitor/hydrogel suspension to each central chamber.
  11. Leave to rest for 5 min.
  12. Top up with additional hydrogel mix to create a meniscus above the chamber and leave to rest for another 5 min.
  13. Carefully add ~30 μL of thrombin across the compartmentalised PDMS array. Do not add directly to the chambers; just apply at the corners. Do this relatively fast as the thrombin acts quickly to polymerise the hydrogel.
  14. Wait a further 5 min.
  15. Now, carefully add 2 mL of skeletal muscle cell growth medium to the dish and place in the incubator.
  16. Wait 24 h after plating the myogenic progenitors, then change the skeletal muscle cell growth medium to skeletal muscle secondary differentiation medium to induce the formation of a 3D sheet of myofibers in the central chamber.
- **Maintenance and optogenetic entrainment (optional) of neuromuscular co-cultures (Figure 4)**
  **Figure 4.**
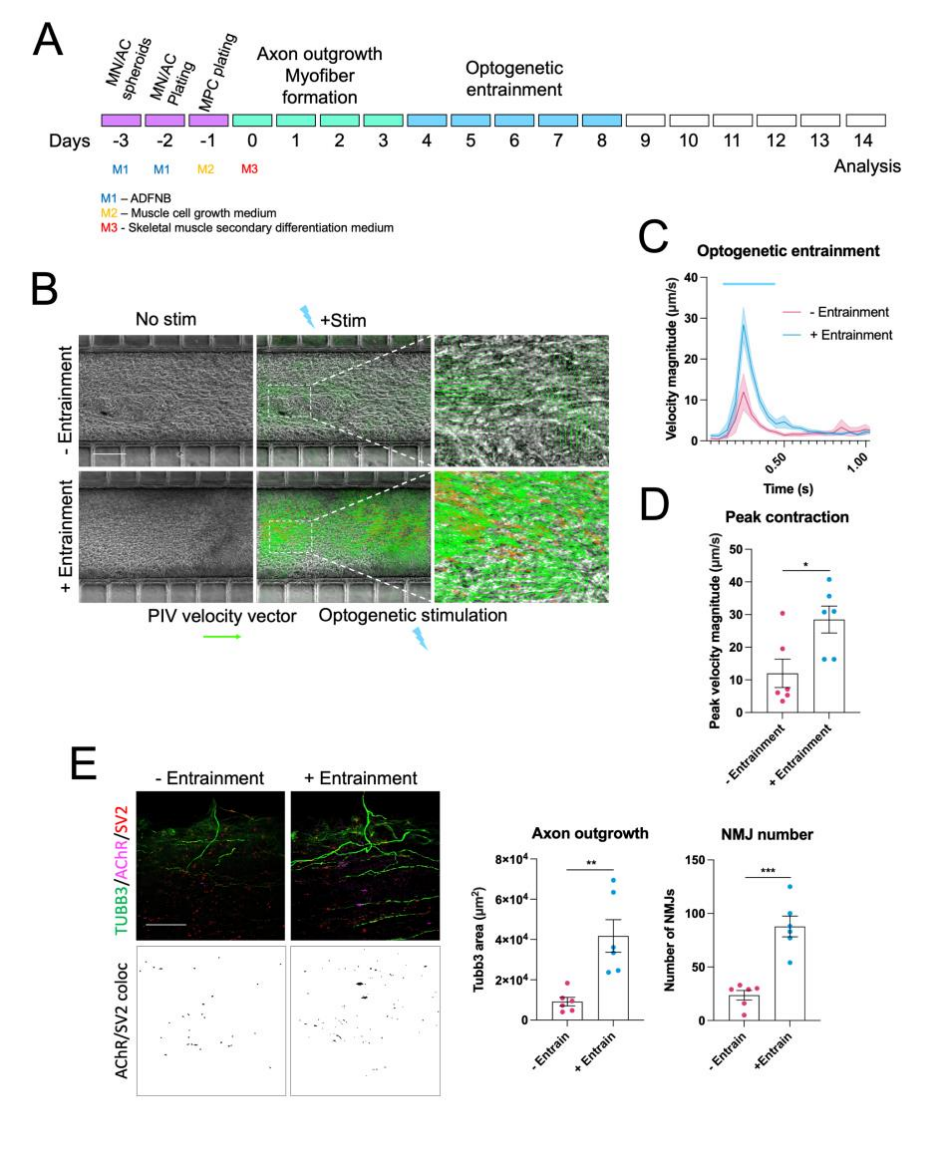
Optogenetic entrainment enhances neuromuscular junction (NMJ) formation and myofiber contractility in human pluripotent stem cell (PSC)–derived neuromuscular circuits. **A.** Schematic showing optogenetic entrainment of wildtype human PSC neuromuscular co-cultures. **B.** Particle image velocimetry analysis of optogenetically evoked myofiber contractions in non-entrained and entrained co-cultures (Scale bar = 200 μm). **C.** Quantification of peak optogenetically evoked myofiber contraction velocity in non-entrained and entrained co-cultures, n = 6. **D.** Immunofluorescence staining for TUBB3, SV2, and AChR alongside SV2/AChR (NMJ) colocalization channels in non-entrained and entrained co-cultures (Scale bar = 50 μm). Quantification of axon outgrowth and NMJ number in non-entrained and entrained conditions, n = 6. Error bars represent the SEM, unpaired non-parametric t-tests used to determine statistical significance. **p < 0.01, ***p < 0.001.
  The optogenetic entrainment section of the protocol is designed for neuromuscular co-cultures containing motor neurons harbouring an optogenetic actuator. In our studies, we used CHR2-YFP stably integrated into the human PSC lines with a PiggyBac transposition system (Harley et al., 2022). Furthermore, this section relies on the use of LED stimulator placed in a tissue culture incubator. In our work, we used a custom built stimulator (Machado et al., 2019), although commercial versions are also widely available. While optogenetic entrainment was not essential to generate functional neuromuscular circuits, it did significantly improve circuit formation and contractility and provides an interesting paradigm to finely control the activity dependence of synapse formation in future studies.
  1. Refresh skeletal muscle secondary differentiation medium every 2–3 days. [Optional]: Combine skeletal muscle secondary differentiation medium with motor neuron medium 50:50 at this stage onwards.
  2. Four days after plating the myogenic progenitors, motor axons should have begun to grow through the microchannels into the central muscle compartment.
  3. Before optogenetic entrainment, add 1:1,000 antioxidant supplement to the 35 mm dish.
  4. Place on top of the LED stimulator in the incubator and stimulate at 20 Hz for 1 h at 40% LED intensity.
  5. After stimulation, completely change the medium to remove toxic free radicals induced by photo-stimulation.
  6. Repeat this for five days.
    Note: The length of entrainment depends on the experimental conditions. We noticed that when treated with drugs that promote myofiber contractility, five days of entrainment would lead to too much detachment, so shorter entrainment periods were used (Paredes-Redondo et al., 2021).
- **Recording and particle image velocimetry (PIV) analysis of optogenetic motor neuron–evoked myofiber contractions (Figures 4A–4D, 5, 6)**
  **Figure 5.**
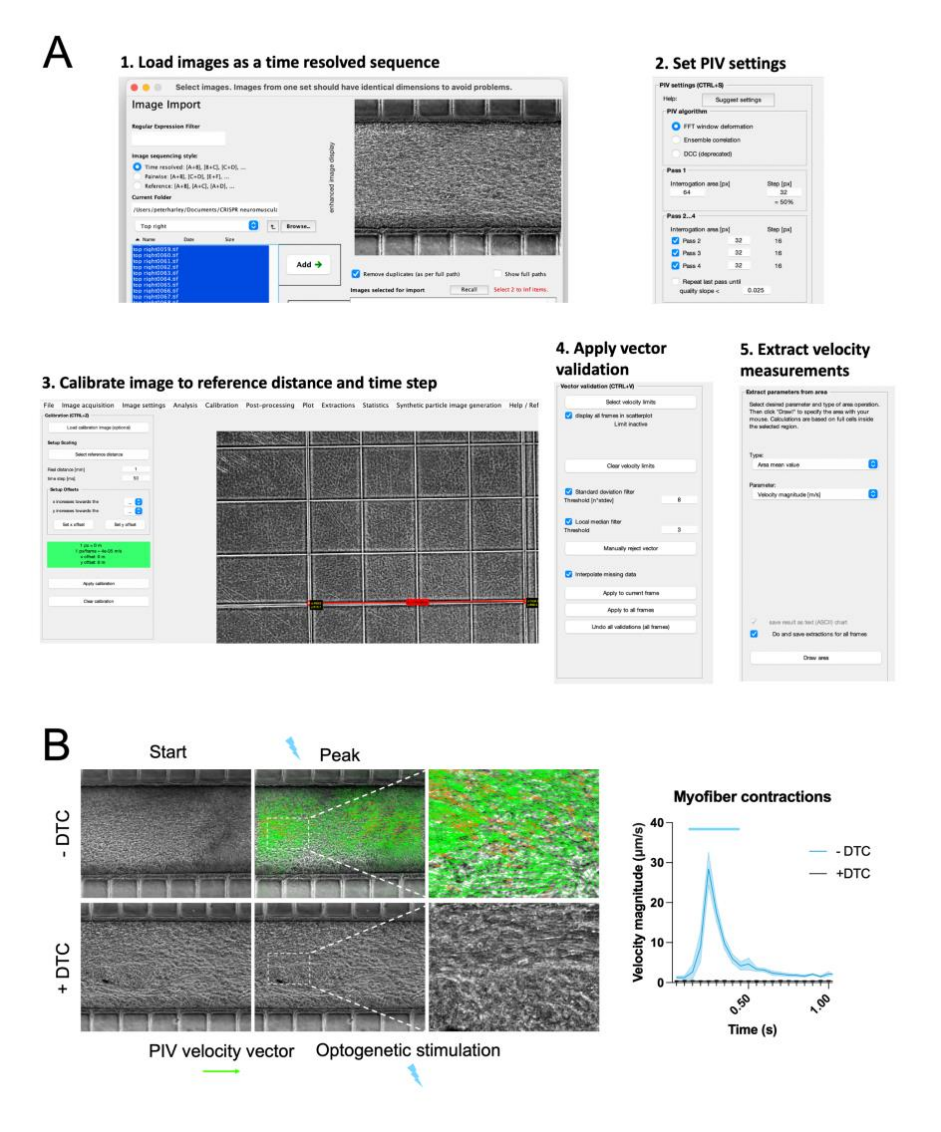
Particle image velocimetry (PIV) analysis of optogenetically evoked myofiber contractions. **A.** PIV analysis steps using the PIVLab Matlab plug-in: 1. Load images as time resolved sequence; 2. Set PIV setting; 3. Calibrate image to reference and time step; 4. Apply vector validation; 5. Extract velocity measurements. **B.** PIV analysis of optogenetically evoked myofiber contractions with and without treatment with the AChR blocker d-Tubocurarine (DTC).
  **Figure 6.**
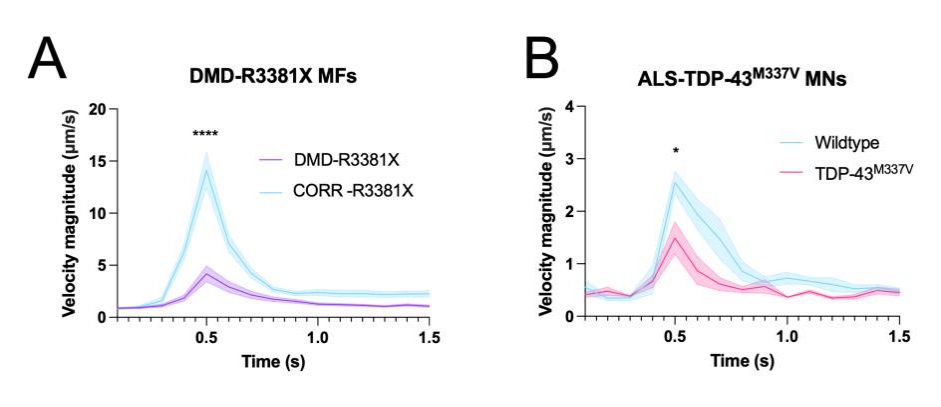
Neuromuscular co-cultures can be used to model amyotrophic lateral sclerosis (ALS) and Duchenne muscular dystrophy (DMD)–related neuromuscular disease phenotypes. **A.** Optogenetically evoked myofiber contractions in neuromuscular co-cultures containing myofibers from DMD patient–derived DMD-R3381X PSCs compared to CRISPR-corrected controls (CORR-R3381X). **B.** Optogenetically evoked myofiber contractions in neuromuscular co-cultures containing motor neurons from ALS patient–derived TDP-43^M337V ^PSCs compared to wildtype controls.
  To briefly test if neuromuscular circuits were functional during the course of the culture, an Olympus X73 microscope with a fluorescent lamp was used to optogenetically stimulate the motor neurons, simply by opening and closing the fluorescent shutter, and to observe associated myofiber contractions. However, for a more controlled analysis, a Thorlabs LED stimulator was used to provide precise optogenetic stimulation via an optical fibre light guide, measured to be 0.2 mW/mm^2^ of light intensity (Paredes-Redondo et al., 2021), which is sufficient to trigger action potentials in CHR2-expressing neurons.
  1. Place the optical fibre coupled with a 470 nm LED light guide >1 cm beneath the 35 mm dish, just out of frame of the microscope’s internal light beam.
  2. Set the LED intensity on the LED driver to 100% and the stimulation interval to 500 ms.
  3. Record 5 s videos at 20 frames per second (FPS) using the CellSens software; manually trigger the LED stimulator during the recording.
  4. Export video recordings as TIFF image sequences.
  5. Download and install the PIVLab Matlab plug-in.
  6. Load images as a time resolved sequence (A + B), (B + C), and (C + D).
  7. In PIV settings, select *FTT* in the *PIV algorithm, Interrogation area* = 64 pixels and *Step* = 32 in *Pass 1*, and *Interrogation area* of 32 px for *Pass 2, 3, and 4*.
  8. Calibrate the image to a reference image with a pattern of known dimensions (for example an image of a hemocytometer chamber taken with the same microscope and settings) and set the time calibration to the interval between pixels, which is 50 ms for a 20 FPS recording.
  9. Click: Analysis > ANALYZE!
  10. For post-processing, apply 8 to *Standard deviation filter* and *Apply to all frames*.
  11. Export results as text (ASCII) chart for all frames as area mean value using the velocity magnitude (m/s) parameter and draw the area of interest for extraction (the central myofiber compartment in this case).
  12. Finally, multiply the velocity magnitude values by 1,000 to derive the velocity magnitude in μm/s.
  13. Multiple recordings of the same conditions are combined by aligning the contractile peaks.
- **Immunohistochemistry and analysis of axon outgrowth and NMJ number/morphology (Figures 4E, 7)**
  **Figure 7.**
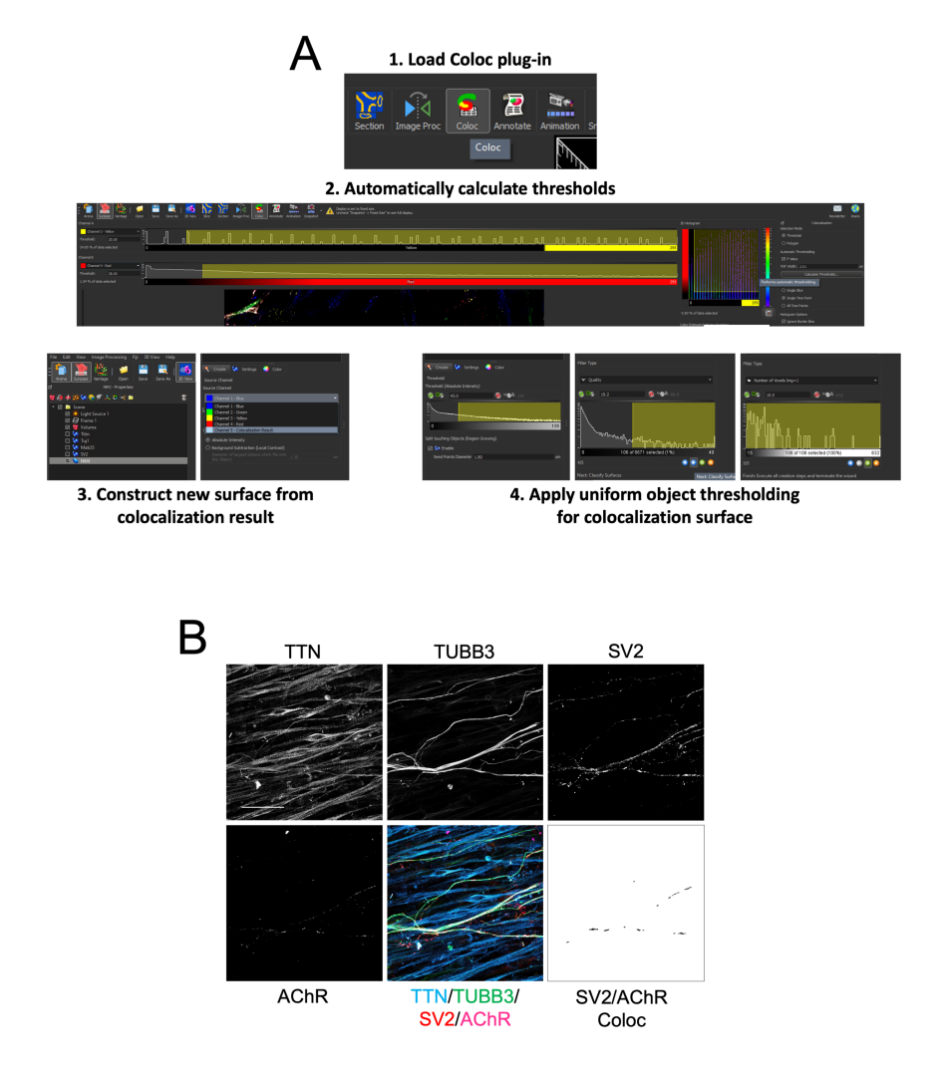
Immunocytochemistry analysis of axonal outgrowth and neuromuscular junction (NMJ) number and morphology. **A.** Steps used in IMARIS software to analyse colocalization of SV2 and AChR objects to infer NMJ number and morphology. **B.** Representative immunofluorescence images for Titin, TUBB3, SV2, and AChR alongside composite images and the colocalization channel for SV2 and AchR (Scale bar = 50 μm).
  1. Following contraction analysis, wash the neuromuscular co-cultures in PBS and fix for 20 min in 4% PFA, 15% sucrose solution.
  2. Wash three times in PBS; then, block in 3% BSA, 0.1% Triton-X-100, and 10% DMSO for 1 h at RT.
  3. Add primary antibodies (Titin 1:20, TUBB3 1:500, SV2 1:200, and AChR 1:100) in blocking solution and leave overnight at 4 °C.
  4. Wash three times in 0.1% PBT and incubate secondary antibodies (1:1,000 dilution) in 0.1% PBT, 10% DMSO overnight at 4 °C.
  5. Wash three times in PBS; then, mount using an 18 mm coverslip and vectashield antifade mounting medium. To mount the samples, add 3–4 drops of mounting medium to the edge of the array, tilt the dish so that the medium flows over the compartments, and then slowly drop the coverslip onto the array, starting with one side.
  6. Image using a confocal microscope over a 40 μm Z-stack at 2 μm intervals.
  7. Using IMARIS image analysis software, create a surface for each channel.
  8. Use the colocalization (Coloc) plug-in to generate a colocalization channel for the SV2 and AChR channels by using the automatic threshold calculation tool.
  9. Create a surface for this colocalization channel.
  10. Now, export object data for each channel, as well as morphology related data, such as area and volume.
  11. Use object number and mean area/volume data from the colocalization channel to infer NMJ number and morphology.
  12. Use sum area/volume data from the TUBB3 channel to infer axonal outgrowth.

## Notes

Owing to the complex and multi-lineage composition of these co-cultures, there is normally a reasonable degree of interexperimental variability in contractility, axonal outgrowth, and NMJ formation. As such, care should be taken to include all appropriate control groups each time co-cultures are generated. Normalisation of data between experiments should also be considered to compare multiple datasets. We recommend that at least four technical replicates are performed for each condition in each experiment, and that the experiments are repeated at least three times to ensure reproducibility. Recommended statistical tests: PIV measurements of myofiber contractions, two-way ANOVA with Sidak’s multiple comparisons test; NMJ number and morphology, one-way ANOVA with Tukey’s or Dunnet’s multiple comparison test.

## Recipes

1. **ADFNB medium (M1)**
  **Table:** 

Reagent	Final concentration	Amount (for 50 mL)
Advanced DMEM/F-12	1 part	23.25 mL
Neurobasal	1 part	23.25 mL
Neurobrew-21	1×	1 mL
N2 supplement	1×	0.5 mL
L-glutamine	2 mM	0.5 mL
Penicillin/streptomycin	1×	0.5 mL
β-mercaptoethanol	1×	50 μL
BSA (5% stock)	0.1%	1 mL
2. **Skeletal muscle secondary differentiation medium (M3)**
  **Table:** 

Reagent	Final concentration	Amount (for 50 mL)
DMEM/F-12	1 part	48.4 mL
N2 supplement	1×	0.5 mL
Insulin-transferrin-selenium	1×	0.5 mL
Penicillin/streptomycin	1×	0.1 mL
L-glutamine	2 mM	0.5 mL
3. **Motor neuron medium (M4) (Optional)**
  **Table:** 

Reagent	Final concentration	Amount (for 50 mL)
DMEM/F-12	1 part	23.75 mL
Neurobasal	1 part	23.75 mL
Neurobrew-21	1×	1 mL
N2 supplement	1×	0.5 mL
L-glutamine	2 mM	0.5 mL
Penicillin/streptomycin	1×	0.5 mL
GDNF	10 ng/mL	5 μL
BDNF	10 ng/mL	5 μL
4. **0.1% PBT**
  **Table:** 

Reagent	Final concentration	Amount (for 500 mL)
PBS	1 part	500 mL
Triton X-100	0.1%	5 mL 10% stock diluted in PBS
